# The impact of time-restricted feeding on energy and macronutrient intake among elite Jordanian football players: a randomized controlled trial

**DOI:** 10.3389/fspor.2025.1657828

**Published:** 2026-01-07

**Authors:** Hadeel Ghazzawi, Razan Mahmoud Omoush, Rand Iblasi, Adam Amawi

**Affiliations:** 1 Department of Nutrition and Food Technology, School of Agriculture, Amman, Jordan; 2 Department of Movement Sciences and Sports Training, School of Sport Sciences, The University of Jordan, Amman, Jordan

**Keywords:** energy, football players, intermittent fasting Jordanian athletes, nutrients, RCT

## Abstract

**Background:**

Nutrition is a key factor in optimizing training, performance, recovery, and health among athletes. Intermittent fasting (IF) is one of the nutritional strategies.

**Objective:**

To compare the effect of an 8-week time-restricted feeding (TRF) protocol vs. a standard diet on energy and macronutrient intake among professional football players.

**Methods:**

A randomized controlled trial was conducted among 30 professional adult male football players, a 16/8 time-restricted feeding (TRF) group vs. a control group. Dietary intake was assessed pre- and post-intervention (12 weeks) using a 7-day food record. Data were analyzed for energy and nutrient intake using ESHA Food Processor® software.

**Results:**

At baseline, both groups consumed less energy and carbohydrates than recommended for elite athletes. Following the 8-week intervention, total energy and macronutrient intakes increased slightly in both groups, but changes were not statistically significant for energy or carbohydrates. The TRF group increased mean energy intake from 33 ± 8.0 to 36 ± 4.9 kcal/kg/day and carbohydrate intake from 4.02 ± 1.48 to 4.27 ± 0.82 g/kg/day, while the Control group increased from 38 ± 12.2 to 42 ± 11.0 kcal/kg/day and from 4.58 ± 2.11 to 5.13 ± 1.73 g/kg/day, respectively. Protein intake significantly decreased within the TRF group (from 2.21 ± 0.60 to 1.84 ± 0.51 g/kg/day, *p* = 0.01), while the Control group showed no significant change. Fat intake increased in both groups but without significant between-group differences. Despite modest improvements, both groups continued to fall below recommended energy and carbohydrate targets, and vitamins D and K remained markedly insufficient post-intervention.

**Conclusion:**

TRF did not significantly improve energy or macronutrient intake compared to the standard diet. Both groups exhibited persistent energy and carbohydrate deficits and inadequate vitamin D and K intake, highlighting the need for structured nutrition support regardless of feeding pattern.

## 1 Introduction

Fasting refers to the voluntary abstinence from caloric intake for a defined period, whereas intermittent fasting (IF) encompasses structured fasting feeding cycles that restrict the timing of food consumption (1). Several forms of IF have been described, including time-restricted feeding (TRF), alternate-day fasting (ADF), alternate-day modified fasting, and periodic fasting (2). One form of IF that has gained tremendous popularity through mainstream media is the so-called time-restricted feeding (TRF). TRF allows subjects to consume *ad libitum* energy intake within a defined window of hours and fasting in the remaining hours (2).

Football, with roughly 265 million registered players, has become the most popular and frequently played sport worldwide in recent decades (3–5). In Jordan, football holds a similarly prominent position, with the national team achieving a historic milestone by qualifying for the 2026 FIFA World Cup and actively participating in regional and international competitions. This reflects the country's growing investment in elite player development and high-performance sports. As the level of competition intensifies globally and locally, the increasing physical and competitive demands of elite football characterized by higher match intensity, frequent fixture congestion, and extended seasons have placed greater emphasis on the role of nutrition in supporting performance and recovery (6). With elite players often competing in over 60 matches per season and facing limited recovery time, dietary strategies must be optimized to support energy availability, enhance recovery, and reduce the risk of fatigue-related injuries (7).

Optimal performance in elite football depends on appropriate dietary strategies due to the sport's demanding physical requirements, including high training volumes, frequent matches, and limited recovery periods (6). Nutrition plays a central role in supporting physiological function, enhancing recovery, and minimizing the risk of injury and illness. International guidelines highlight the importance of nutrient intake's quantity, composition, and timing to maximize training outcomes and overall performance (8). While general recommendations for athletes are available, football-specific dietary requirements differ based on training intensity, match frequency, positional demands, and individual variation. However, there remains a lack of comprehensive, evidence-based dietary guidelines explicitly tailored to football (6, 9, 10). Energy requirements for elite football players typically range from 40 to 70 kcal/kg/day, depending on training intensity and individual demands (11). Balancing energy intake with expenditure is essential to prevent energy deficits or surpluses, which can negatively impact performance and recovery. Therefore, Dietary strategies must be adjusted according to fluctuating training loads (6, 12). Carbohydrates are the primary fuel source for high-intensity exercise, supporting muscle contraction, central nervous system function, and glycogen replenishment. Recommended carbohydrate intake ranges from 5 to 10 g/kg/day, with UEFA suggesting 6–8 g/kg/day during congested fixture periods (12). Protein intake, typically 1.2–2.0 g/kg/day, is essential for muscle repair, adaptation, and maintaining structural tissues, including tendons and bones, Protein targets for football players were set at 1.6–1.8 g/kg/day, which aligns with ISSN and UEFA nutritional recommendations for team-sport athletes (6, 13). Dietary fat should account for 20%–35% of total energy intake, contributing to the absorption of fat-soluble vitamins and supplying essential fatty acids such as omega-3s (10). In football players, inadequate key micronutrients such as iron, vitamin D, calcium, and B vitamins may impair muscle function, bone health, and energy metabolism. A well-balanced diet that ensures adequate macronutrients and micronutrients while maintaining energy balance is fundamental to optimizing performance, supporting recovery, and reducing the risk of illness and injury in elite football players (11).

In athletes, including football players, TRF may contribute to improved body composition, enhanced metabolic flexibility, and reductions in inflammation and oxidative stress, all of which are relevant for performance and recovery (14, 15). Several studies suggest that TRF can lead to a spontaneous reduction in energy intake and may improve insulin sensitivity, lipid profiles, and circadian rhythm alignment (14). However, the application of TRF in football presents unique challenges. Given the sport's high-intensity, intermittent nature and demanding training schedules, there is concern that restricted feeding windows may compromise energy availability, delay muscle recovery, or result in inadequate nutrient intake, particularly for carbohydrates and protein (16, 17). Football players require precise timing of macronutrient intake to support glycogen replenishment, muscle repair, and adaptation, especially during periods of high training load or match congestion (8, 16). Therefore, while TRF may offer health-related benefits, its implementation in football must be carefully managed to ensure that dietary needs are met within the limited feeding window. Individualized planning, nutrient-dense meal choices, and strategic timing are essential to integrate TRF without compromising performance or recovery.

Greater attention must be directed toward optimizing the dietary intake of football players in Jordan, as this critical period significantly influences performance and recovery. To address this gap, the present study aimed (1) to evaluate the effect of an 8-week time-restricted feeding (TRF) protocol on energy and macronutrient intake among elite football players, and (2) to assess the adequacy of micronutrient intake relative to recommended reference values for team-sport athletes.

## 2 Materials and methods

### 2.1 Participants

This randomized controlled trial (RCT) included thirty male football players were chosen from JFA (Jordan Football Association) and collection of the sample was taken during both pre-season and in-season from July to October. These players ranged in age from 19 to 35. Physiological exercises (sprinting, alternating stamina) were strategic throughout each training session. Inclusion was limited to male football players, Jordanian nationality, exhibiting more than 5 years of continued experience. Female athletes were not included due to physiological differences associated with the menstrual cycle, particularly fluctuations in cortisol and testosterone levels across different phases, which could potentially influence dietary intake and metabolism (11, 18, 19). Exclusion criteria included active smoking (cigarettes, pipes, cigars, and e-cigarettes), diagnosed metabolic diseases, cardiovascular or respiratory disorders, and orthopedic issues within the past 5 years that could have limited exercise performance. Football players had not experienced significant weight loss (≥10% of body weight) in the 6 months prior to enrollment and were not practicing any form of intermittent fasting. Additionally, individuals who used anabolic steroids, or took medications (e.g., steroidal and non-steroidal) or dietary supplements (such as creatine, beta-alanine) that might have interfered with study outcomes were excluded from participation. In accordance with the approved research design authorized by the Faculty of Graduate Studies, the Department of Scientific Research, and the Institutional Review Board (IRB) at the University of Jordan (Decision Code: 250/2024), submitted by Dr. Hadeel Ali Ghazzawi from the School of Agriculture, written informed consent was obtained from each football player who participated in the study.

### 2.2 Anthropometric measurements

The bioelectrical impendence (BIA) was measured by an InBody 230 device (InBody Co., Ltd., Seoul, KOREA). Validity testing in healthy adults demonstrated strong agreement with DXA for whole-body fat mass, percent fat, and fat-free mass (*r* = 0.94–0.99), with minor biases in segmental measurements, and has a standard error of estimate (SEE) of ±3% (20). A cohort of elite football players also observed high reliability and low measurement error (21, 22). All the anthropometric and BIA measures were carried out at the team camp. In this research, lean body mass index (LBM), percent of body fat (BFP), and body weight were the BIA measures that were used. Body weight measurements were obtained by football players wearing minimal clothing and being barefoot. Readings were obtained within 0.1 kg of the closest unit. The subjects' heights were measured using a SECA (Wall Mounted Portable Stadiometer) digital land scale (Model 876) to the closest half-centimeter. All the measures were carried out in the morning, following a fasting period of 8–10 h.

### 2.3 Dietary intake measurements

Dietary intake was comprehensively assessed Pre- and Post-intervention using a 7-day food record (FR). During the 8-week intervention, weekly 3-day food records, coupled with daily telephone interviews and a mobile application, were employed solely to monitor protocol adherence and compliance for both groups. As baseline assessments were conducted during the preparatory (pre-season) phase in August, and post-intervention assessments were completed during the competitive (in-season) phase (September–October). The study therefore spanned both training periods. To ensure nutritional adequacy and control for variations in workload, individualized meal plans were developed and updated weekly according to each player's estimated energy requirements and training intensity. Estimated energy requirements (EER) increased by 6%–10% from the pre-season to in-season period following adjustments for training intensity, match congestion, and recovery demands. Despite these controls, the transition between training phases may have influenced dietary intake patterns.

**Figure 1:**
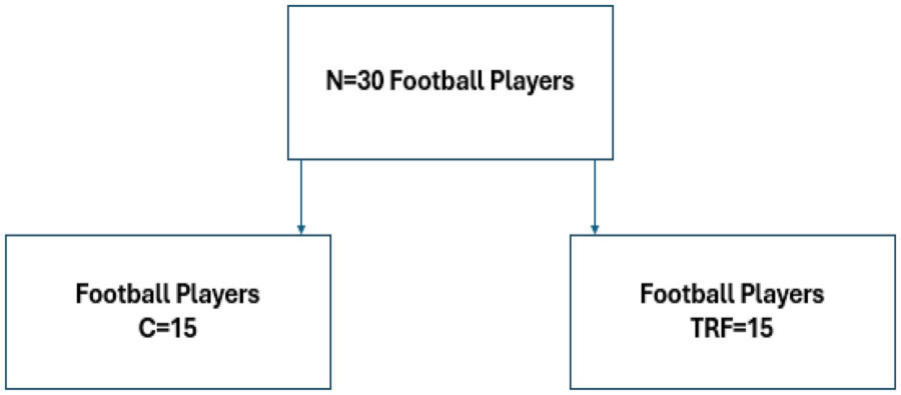
Experimental layout. Time restricted feeding (TRF), standard diet (C).

Dietary intake was analyzed using the Dietary Processor® Nutrition Analysis ESHA program (Ver 10.9/2011). We determined the total amount of calories consumed and the composition of carbohydrates, fats, proteins, and micronutrients in football players' diets. Before starting the trial, football players were given written instructions about the time and the number of meals they consumed. Using a customized guidebook, they were provided with detailed instructions on determining the appropriate portion sizes and identifying all food and drink consumption. To enhance accuracy, each participant was also given a set of measuring cups to assist in quantifying their intake. A telephone number was accessible to all football players if they had any inquiries. Immediately after the end of the study, the food records were thoroughly examined. A demographic form collected detailed information on football players' typical eating habits and related behaviors, including nutritional supplements, dietary preferences and aversions, and weight-control practices. Pre-intervention, all football players underwent baseline assessments of dietary intake and anthropometric measurements. Following this, thirty football players were randomly assigned to either a time-restricted feeding group (TRF; *n* = 15) or a control group (CON; *n* = 15) using a random number table. The experimental layout is displayed in (Figure 1). The control group followed a standard diet, while the TRF group adhered to a time-restricted feeding protocol. All meal plans for both groups were designed by a registered dietitian and updated weekly to be isoenergetic, targeting the Estimated Energy Requirement (EER) for elite football players, therefore meal plans tailored to their respective energy requirements, training intensity, and match schedule. The primary distinction between the groups was that the TRF group consumed all meals and snacks within an 8-hour window (12:00–20:00), All training sessions were conducted between 16:00 and 18:00, and the TRF feeding window was designed accordingly to ensure participants could consume meals both before and after training within the restricted period. While the Control (CON) group followed the same dietary composition and energy targets but without any meal timing restrictions.

### 2.4 Interventions and protocols

The intervention lasted 8 weeks, conducted between August and October 2023, covering the transition from the pre-season preparatory phase to the early in-season competitive period. Baseline measurements were obtained at the end of pre-season training, and post-intervention assessments occurred during the early in-season phase. The study design was structured to maintain consistency in training frequency and load across both phases.

TRF interventions followed a 16/8 time-restricted feeding protocol. All football players consumed two to three meals during 8 h (between 12 and 8 p.m.). The remaining 16 h per 24-hour period will constitute the fasting period, during which football players were only allowed to consume water, tea, and coffee (without caloric additives). The non-TRF diet corresponding to the football players was the same meal plan without any timing restrictions, and they ingested their caloric intake as three meals and snacks consumed at 8 a.m., 1 p.m., and 8 p.m.

In both groups, the distribution of macronutrients was the same. Every participant received a meal plan based on their needs using the Harris-Benedict equation. The ISSN recommends that energy requirement is 40–70 kcal/kg/day, protein ingestion is 1.6–1.8 g/kg of body mass, carbohydrate ingestion is 6–10 g/kg, and lipid intake is 20%–35% (10). The distribution of calories for the TRF group was 54%, 18%, and 28% at 12 p.m., 4 p.m., and 8 p.m., respectively, while the CON group consumed 54%, 18%, and 28% of their daily calories at 8 a.m., 1 p.m., and 8 p.m., respectively. The sample meal plan is shown in Appendix A. During training and compliance logs, adherence to the fasting protocol was monitored using weekly dietary records for 3 days (2 weekdays and 1 weekend). Telephone interviews were conducted every day of recording to ensure the football players' information was accurate and to obtain the details rapidly before football players failed to remember these data. Participant compliance data were analyzed. In addition, the mobile application was used to register meals and the amount of food ingested accordingly.

### 2.5 Statistical analysis

Data were analyzed using SPSS version 17.0 (Chicago, IL). Normality was verified using the Shapiro–Wilk test. A two-way mixed-design ANOVA assessed the interaction between time (pre- vs. post-intervention) and group (TRF vs. CON) for each dependent variable (energy, macronutrients, and micronutrients). *Post hoc* paired-sample *t*-tests were applied for within-group comparisons when significant interactions were observed. Independent-sample *t*-tests compared baseline values between groups. Statistical significance was set at *p* < 0.05.

## 3 Results

### 3.1 Characteristics of sample

The anthropometric characteristics of the participants at baseline are presented in Table 1, with values reported as mean ± standard deviation. Fat mass and muscle mass were estimated using bioelectrical impedance analysis (BIA).

**Table 1:** Baseline characteristics of participants by study group (mean ± SD).

Anthropometric indices	TRF (*n* = 15)	CON (*n* = 15)	*p*-value
Age (years)	22.5 ± 2.8	22.8 ± 2.9	0.74
Weight (kg)	176.6 ± 4.5	177.3 ± 4.7	0.68
Height (cm)	73.5 ± 6.9	72.9 ± 7.3	0.82
Fat (kg)	9.8 ± 2.1	9.5 ± 2.4	0.79
Fat (%)	13.4 ± 2.1	13.1 ± 2.4	0.77
Muscle mass (kg)	36.9 ± 2.8	37.1 ± 2.5	0.84
Muscle mass (%)	50.4 ± 2.6	50.7 ± 2.8	0.88

Body-composition values were obtained using BIA (InBody 230). Standard error of estimate (SEE): ±3% for body fat and ±2.1 kg for lean mass.

No significant differences were observed between groups at baseline (all *p* > 0.05).

Post-intervention anthropometric measurements showed no significant changes in body weight or lean mass (*p* > 0.05).

### 3.2 Dietary intake

Pre-intervention and during the training period, protein intake was significantly higher in the Control group compared to the TRF group (*p* = 0.03), while no significant differences were observed between groups for total energy intake, carbohydrate (CHO), or fat intake (Table 2). Post-intervention, carbohydrate intake increased modestly in both groups (*p* > 0.05). Protein intake significantly decreased within the Control group (*p* = 0.03) but remained unchanged in the TRF group (*p* > 0.05). Fat intake increased in both groups, though changes were not statistically significant. However, no significant differences in total energy, CHO, and fat intake were found between groups.

**Table 2:** Calorie and macronutrient intake values among football players during the training period.

Nutrient	Prescribed target	Dietary intake (*M* ± SD) pre	Dietary intake (*M* ± SD) post	RV	*p*-value
Average intake (kcal/day)					
TRF	—	2,502 ± 539.47	2,735 ± 280	-	0.49
CON		2,890 ± 815.33	3,240 ± 792.78		
Energy (kcal/kg/day)					
TRF	45 ± 5	33 ± 8.04	36 ± 4.87	40–70^a^	-
CON		38 ± 12.18	42 ± 11.01		
CHO. (g/kg/day)					
TRF	6–8	4.02 ± 1.48	4.27 ± 0.82	5–10^b^	0.49
CON		4.58 ± 2.11	5.13 ± 1.73		
CHO. (total intake/day)					
TRF		307 ± 102.41	326 ± 58.93		0.49
CON		350 ± 138.13	392 ± 123.29		
CHO. (%)					
TRF	55–60	48%	48%	50%–60%	
CON		48%	48%		
Dietary prot. (g/kg/day)					
TRF	1.6–1.8	1.73 ± 0.27	1.73 ± 0.27	1.2–2^c^	0.03*
CON		1.84 ± 0.51	1.71 ± 0.42		
Dietary prot. (total intake/day)					
TRF		132 ± 35.46	132 ± 14.57	-	0.03*
CON		169 ± 42.03	141 ± 34.86		
Dietary prot. (%)					
TFR	15–20	21%	19%	15%–20%	
CON		22%	17%		
Dietary fat (g/kg/day)					
TRF	1.0–1.2	1.19 ± 0.40	1.43 ± 0.40		0.67
CON		1.29 ± 0.30	1.72 ± 0.55		
Dietary fat (total intake/day)					
TRF		91 ± 30.98	109 ± 26.67	-	0.67
CON		98 ± 23.32	131 ± 42.74		
Dietary Fat (%)					
TRF	25–30	31%	33%	25%–35%^d^	-
CON		30%	35%		

SD, standard deviation. Values are presented as mean ± standard deviation (SD). *P*-values represent within-group comparisons (pre vs. post). Between-group differences in change scores are reported in the text. TRF, time-restricted feeding; CON, control group.

a Recommended values (RV) are based on the International Society of Sports Nutrition (ISSN) consensus statement (11), ISSN/IOC/ACSM (6, 17, 23).

b (10, 24, 25).

c (7).

d (11).

* Indicates statistical significance at *p* ≤ 0.05.

Macronutrient intake was further categorized according to whether it fell below, within, or above the recommended values set by the International Society of Sports Nutrition (ISSN). Table 3 presents the distribution of participants across these categories at both pre- and post-intervention time points for total energy, carbohydrate (CHO), protein, and fat intake.

**Table 3:** The numbers and percentages within the football players consuming macronutrients above or below the recommendations pre-intervention and post-intervention.

Micronutrients	Lower % (*N*)	Lower % (*N*)	Within % (*N*)	Within % (*N*)	Higher % (*N*)	Higher % (*N*)
	Pre	Post	Pre	Post	Pre	Post
Calorie intake	58% (15)	30% (8)	42% (11)	66% (17)	-	4% (1)
CHO	73% (19)	50% (13)	23% (6)	50% (13)	4% (1)	-
Protein	8% (2)	42% (11)	54% (14)	58% (15)	38% (10)	42% (11)
Fat	70% (18)	4% (1)	30% (8)	73% (19)	-	23% (6)

Recommended values compared to ISSN; L, football players with intakes lower than the ISSN; W, football players with intakes within the ISSN; H, football players with intakes higher than the ISSN; *N* represents the number of football players; % Represents the percentage of football players. SD, standard deviation. Data represent the percentage (and number of participants) whose intakes fell below (“Lower”), met (“Within”), or exceeded (“Higher”) ISSN recommendations. TRF, time-restricted feeding; CON, control.

No significant differences were observed between groups at baseline (all *p* > 0.05).

Table 4 shows micronutrients intake for football players in pre and post-intervention, pre- intervention the TRF group had lower-than-recommended intakes of several micronutrients, including Vitamin A, Biotin, Vitamin D, Vitamin E, Vitamin K, Vitamin B5, Calcium, Choline, Iodine, Magnesium, Potassium, and Zinc. In contrast, the control group showed inadequate intake of biotin, vitamin D, cholesterol, vitamin K, potassium, and iodine. Post-intervention, the TRF group exhibited significant improvements in most micronutrient intakes (*p* < 0.05), except for Vitamin B6 and Manganese, which remained in normal ranges.

**Table 4:** Micronutrients intake pre- and post-intervention compared to recommended values.

Nutrient	Mean intake (pre) ± SD	Mean intake (post) ± SD	EAR-UL	*p*-value between groups (TFR vs. CON)	*p*-value within group (pre vs. EAR-UL)	*p*-value within group (post vs. EAR-UL)
Vit A (mcg)						
TRF	175.30 ± 97.97	776.47 ± 906.80	625–3,000	0.91	0.00*	0.00*
CON	1,138.55 ± 3,160.20	343.81 ± 116.84				
B1 (mg)						
TRF	2.19 ± 1.66	3.38 ± 1.03	0.8–1.2**	0.69	0.00*	0.16
CON	1.98 ± 0.87	5.35 ± 4.76				
B2 (mg)						
TRF	2.41 ± 1.85	4.03 ± 1.44	1.1–1.8	0.52	0.05*	0.00*
CON	2.32 ± 1.69	5.72 ± 5.18				
Niacin eq (mg)						
TRF	27.58 ± 8.96	41.64 ± 9.11	12–35	0.48	0.00*	0.69
CON	38.67 ± 16.80	35.26 ± 6.91				
B5 (mg)						
TRF	4.88 ± 1.87	7.15 ± 1.31	5**	0.6	0.08	0.12
CON	10.51 ± 8.11	5.87 ± 1.38				
B6 (mg)						
TRF	5.03 ± 12.44	2.18 ± 0.69	1.1–100	0.67	0.00*	0.3
CON	3.49 ± 2.99	1.94 ± 0.44				
B12 (mcg)						
TRF	2.09 ± 0.90	8.00 ± 8.81	2–2.4	0.37	0.09*	0.00*
CON	12.55 ± 24.28	3.48 ± 1.54				
Biotin (mcg)						
TRF	16.62 ± 8.28	31.89 ± 11.44	30**	0.6	0.00*	0.00*
CON	22.14 ± 10.36	24.95 ± 10.57				
Vit C (mg)						
TRF	77.21 ± 42.60	182.70 ± 68.37	75–2,000	0.19	0.12	0.00*
CON	166.10 ± 124.90	146.64 ± 64.48				
Vit D (mg)						
TRF	0.92 ± 0.90	1.86 ± 1.25	10–100	0.00*	0.43	0.27
CON	1.12 ± 0.02	1.32 ± 1.15				
Vit E (mg)						
TRF	5.44 ± 3.25	10.76 ± 4.53	12–1,000	0.47	0.00*	0.00*
CON	26.81 ± 73.90	7.61 ± 1.99				
Folate (mcg)						
TRF	410.75 ± 197.18	571.90 ± 113.89	320–1,000	0.13	0.02*	0.26
CON	533.84 ± 199.39	519.31 ± 117.54				
Vit K (mcg)						
TRF	43.70 ± 35.05	72.08 ± 41.54	120**	0.02*	0.00*	0.00*
CON	80.89 ± 72.38	51.32 ± 15.86				
Choline (mg)						
TRF	139.86 ± 91.30	325.13 ± 142.95	550–3,500**	0.84	0.00*	0.00*
CON	253.09 ± 212.07	223.50 ± 128.98				
Calcium (mg)						
TRF	436.35 ± 207.30	832.78 ± 346.40	800–2,500	0.86	0.00*	0.00*
CON	843.68 ± 501.63	549.90 ± 190.20				
Cupper (mg)						
TRF	0.91 ± 0.37	1.97 ± 1.46	0.7–10	0.06	0.00*	0.06
CON	2.59 ± 4.84	1.17 ± 0.31				
Iodine (mcg)						
TRF	61.16 ± 45.31	126.40 ± 42.68	95–1,100	0.26	0.00*	0.43
CON	79.17 ± 44.98	105.12 ± 56.83				
Iron (mg)						
TRF	18.00 ± 10.68	22.98 ± 6.84	6–45	0.61	0.00*	0.01*
CON	20.35 ± 7.98	17.66 ± 2.26				
Magnesium (mg)						
TRF	214.36 ± 84.48	336.88 ± 86.20	330–350	0.6	0.00*	0.00*
CON	367.05 ± 176.81	276.59 ± 61.23				
Manganese (mg)						
TRF	12.95 ± 27.00	5.16 ± 2.29	2.3–11**	0.06	0.00*	0.00*
CON	4.43 ± 2.75	3.90 ± 0.10				
Phosphorus (mg)						
TRF	737.29 ± 287.53	1,267.97 ± 294.73	580–4,000	0.07	0.00*	0.02*
CON	1,035.25 ± 522.47	1,008.12 ± 229.22				
Potassium (mg)						
TRF	1,974.37 ± 630.34	3,598.65 ± 856.65	3,400**	0.49	0.00*	0.02*
CON	2,907.17 ± 756.91	2,962.09 ± 762.55				
Selenium (mcg)						
TRF	78.75 ± 28.31	121.28 ± 42.09	45–400	0.16	0.00*	0.04
CON	105.23 ± 59.83	89.41 ± 31.00				
Sodium (mg)						
TRF	2,371.74 ± 645.17	3,448.51 ± 896.65	1,500–2,300**	0.24	0.01*	0.00*
CON	3,173.78 ± 733.99	2,698.40 ± 714.99				
Zinc (mg)						
TRF	7.71 ± 2.73	11.82 ± 2.57	9.4–40	0.33	0.00*	0.05*
CON	19.12 ± 14.93	9.25 ± 2.25				

*P* (TRF vs. CON) compares the two groups at the same time point. “*P* (pre vs. EAR–UL)” and “*p* (post vs. EAR–UL)” compare each group's mean intake to the recommended range (EAR–UL) before and after the intervention.

*Indicates statistical significance at *p* ≤ 0.05, and ** indicates statistical significance at *p* ≤ 0.01.

Conversely, the Control group experienced a decline in multiple micronutrients, including Vitamin A, Niacin, Vitamin E, Vitamin B5, Vitamin B6, Vitamin B12, Vitamin C, Folate, Choline, Vitamin K, Calcium, Copper, Iron, Magnesium, Manganese, Phosphorus, Sodium, Selenium, and Zinc. Improvement within groups was observed in post-intervention intakes of Vitamin A, Vitamin B2, Vitamin B12, Biotin, Vitamin C, Iron, Phosphorus, Vitamin E, Choline, Calcium, Magnesium, Manganese, Potassium, Sodium, and Zinc. Despite general improvements in the TRF group, both groups' Vitamin K and Vitamin D remained below recommended levels.

## 4 Discussion

The Jordanian national football team has demonstrated notable progress and competitiveness on the international stage, reaching the finals of the AFC Asian Cup and recently qualifying for the FIFA World Cup 2026, marking historic achievements for the country. This study is part of a larger research project investigating various factors influencing athletic performance. The next phase will examine the effects of intermittent fasting on body composition, selected hormone levels, and physical performance, providing a comprehensive understanding of key elements impacting the success of elite Jordanian football players.

Proper nutrition is essential for optimal athletic performance. This study examines these athletes' energy, carbohydrate, protein, and fat intake, identifying potential deficiencies while acknowledging that intake levels may be adequate in some instances (26). These findings emphasize the need for targeted dietary interventions to enhance performance and overall health. Furthermore, to the best of our knowledge, no studies have been carried out to evaluate the dietary consumption of Jordan football players, which indicates that further study is required.

Burke et al. recommend that football athletes eat adequate energy to engage in physical activity, improve their fat-free body weight, and decrease the amount of fat in their body mass (12). The guidelines state that the energy need of an athlete is between 40 and 70 kcal/kg of body mass. Our research found that the average amount of energy that football players consumed daily was 2,695 ± 640 kcal, equivalent to 35.30 ± 9.94 kcal per kilogram of body mass (Table 2). These values are lower when compared to the results for athletes from various countries like the Netherlands (2,988 ± 583 kcal per day and 38.8 ± 7.6 kcal per kilogram of body mass per day) (27), a group of Brazilian professional football players with a daily calorie of 40.74 ± 12.81 kcal) (28), football athletes in the Dutch Premier League (3,285 ± 354 kcal per day; 42.4 ± 3.5 kcal per kilogram of body mass per day) (29), the professional junior players in Spain (3,003 calories) (30), Olympic athletes from Puerto Rico (3,952 calories) (31), Italian professionals (3,650 calories) (32), top Swedish athletes (4,929 calories), and professional athletes from Italy (33), as well as Danish players (3,738 kcal) (34), and Greek footballers who play professionally (3,442 ± 158 kcal/day; 46 ± 2.1 kcal/kg body weight/day) (35) were analyzed. These discrepancies may be attributed to differences in athletes' body sizes, training statuses, skill levels, and the methods used to assess dietary intake across studies.

TRF did not significantly change energy or macronutrient intake compared to the control group. This indicates that restricting the feeding window, by itself, neither improved nor impaired dietary adequacy under real-world training conditions. These findings align with Burke et al. (36), who note that free-living athletes often struggle to meet energy requirements due to fluctuating training loads and challenges in estimating exercise energy expenditure.

While it is often assumed that highly trained athletes can easily replace expended energy, this process is frequently hindered by both physiological and behavioral factors. Following intense training, athletes often experience exercise-induced appetite suppression, making it difficult to consume the large volume of food necessary to meet their Estimated Energy Requirements (EER) in short intervals (37). During extended training days with limited breaks, this reduced appetite coupled with insufficient time available for planned meals can lead to poor compliance, meal skipping, or compensatory eating of convenient, but often nutrient-poor, energy-dense foods (Caruana and colleagues, reference needed). These behaviors collectively contribute to the chronic low energy availability observed in our players, regardless of their time-restricted feeding status (38).

The average energy intake observed in our study (∼35 kcal/kg/day) was markedly below the 40–70 kcal/kg/day recommended for elite football players, indicating low energy availability consistent with the diagnostic threshold for RED-S. Such deficits can impair bone health, endocrine function, and recovery capacity, emphasizing the need for systematic nutrition monitoring in this population. Nonetheless despite apparent dietary insufficiency relative to the Harris-Benedict predicted EER, body weight and lean mass did not significantly change, suggesting that the equation may have overestimated true energy requirements in this sample an observation reported previously in elite football players during early in-season phases. Yet these challenges in meeting energy requirements can contribute to Relative Energy Deficiency in Sport (RED-S), a condition characterized by insufficient energy availability to support optimal physiological function (39). RED-S can impair athletic performance and recovery and increase the risk of injury and illness, underscoring the critical importance of adequate dietary intake in athletes undergoing intense training schedules (40), while energy intake fell below calculated requirements, no reductions in body weight or lean mass were observed, suggesting that the RED-S risk indicated by dietary data did not manifest physiologically during our intervention.

Although athletes are generally expected to meet their energy and nutrient requirements through a well-balanced diet, achieving this can be challenging due to the intensity and duration of training (38). Post-exercise appetite suppression, limited time between meals, and congested training schedules often hinder adequate energy intake (37). Additional barriers such as limited access to qualified sports nutritionists, buffet-style meal arrangements, financial constraints, and insufficient nutritional knowledge further compromise dietary adequacy (41, 42). Moreover, many athletes engage in suboptimal eating practices, including meal skipping and the consumption of inappropriate foods around training and competition, which can impair energy replenishment and recovery processes (43). Persisting with such dietary habits and lifestyle behaviors may exacerbate macronutrient and micronutrient deficiencies, ultimately compromising athletic performance and recovery. Therefore, implementing evidence-based and structured nutritional interventions is essential to address these challenges and support the health and performance of athletes.

Our research found that the average amount of CHO that TRF consumed daily on training day after administration of dietary intervention was 326 ± 58.93 g/d (48%), equivalent to 4.27 ± 0.82 g/kg/d. As presented in (Table 2), CHO intakes were improved in the TRF group compared to the control, but it was not statistically significant. However, there was a slight decrease in CHO intake in the control group. When the data were compared to the norms that had been established, it was revealed that the intake was not sufficient to fulfill the suggested consumption levels for athletes who participated in daily exercise for roughly 4 h. The percentage of football players consuming carbohydrate intake that was lower than recommended dropped significantly from 73% to 50%. In comparison, those within the recommended range doubled from 23% to 50%, indicating a more balanced intake distribution (Table 3).

The TRF group derived an average of 48% of their energy from carbohydrates in the training phase, which is lower than reported for Italian (55.8%) and Puerto Rican (53.2%) athletes but slightly higher than Swedish (47%) and Danish (46.3%) players (31–34). However, the actual carbohydrate intake was 4.27 g/kg/day, substantially below the recommended 7–8 g/kg/day for maintaining glycogen stores during training and competition, according to the American College of Sports Medicine and ADA (44). This intake also falls short of the 5–10 g/kg/day range specifically recommended for football players, indicating a significant deficiency in carbohydrate consumption among football players (6, 9, 10).

Pre-intervention, carbohydrate intake accounted for 48% of total energy, below the recommended >55% for football players and lower than the 52%–56.6% range reported in previous studies. Despite an increase in absolute carbohydrate intake post-intervention, the percentage remained unchanged at 48%. This aligns with other studies showing male football players often consume <50% of their energy from carbohydrates (8, 27, 29, 30, 45–47).

During the training phase, 50% of the athletes consumed carbohydrates below the recommended 57 g/kg/day despite training for approximately 4 h daily. This insufficient intake may impair performance by limiting glycogen availability, a key factor in delaying fatigue and sustaining high-intensity efforts (48). Starting matches with low glycogen stores has been linked to reduced performance, particularly in the second half (49). The study also found that athletes did not periodize carbohydrate intake according to training intensity, potentially due to hormonal changes from intense exercise that suppress appetite. High-intensity physical exercise may affect the amount of hormones, which include ghrelin, glucagon-like peptide one (GLP-1), pancreatic polypeptide (PP), and YY peptides (PYYY), according to the findings of the research. These hormones are the ones responsible for regulating hunger (50). These findings highlight the need for targeted nutritional education for athletes and coaching staff to optimize carbohydrate consumption and support performance and recovery (33).

Post-intervention, the TRF group consumed an average of 132 ± 14.57 g/day of protein (1.73 ± 0.27 g/kg/day), as presented in (Table 2), aligning with the control group, which improved due to nutritional education and follow-up. This intake falls within the recommended ranges for football players (1.2–2.0 g/kg/day), especially during intense training periods (26). While intakes above 1.7 g/kg/day may not enhance post-exercise muscle synthesis, they could support body composition goals (32, 33). Compared to prior studies, the protein intake observed here is similar to that reported by Kirwan et al. (1.8 g/kg/day) and slightly lower than values reported by Abbey et al. (2 g/kg/day) (51, 52). These findings suggest the TRF group achieved adequate protein intake for recovery and performance without exceeding optimal thresholds.

Following the dietary intervention, the TRF group consumed an average of 109 ± 26.67 g/day of fat (1.43 ± 0.40 g/kg/day), as presented in (Table 2), accounting for 33% of total energy intake within the recommended range of 25%–35% set by ACSM and ISSN (11). Although both groups showed increased fat intake, the change was not statistically significant. Adequate fat consumption is essential for absorbing fat-soluble vitamins, supporting hormone levels, and maintaining overall health. However, when combined with low-calorie intake, inadequate carbohydrate intake may contribute to negative energy balance and impaired athletic performance, consistent with prior research findings (12, 34, 53, 54).

This study highlights the significant role of micronutrient intake in supporting the health and performance of professional football players. Pre-intervention data revealed inadequate intake in key micronutrients among the TRF group, particularly in vitamins A, D, E, K, C, and B5, Choline, and minerals like calcium, Iodine, potassium, Zinc, and magnesium, as presented in (Table 4). While the dietary intervention improved most micronutrient levels in the TRF group, vitamins B6 and manganese remained low, though within normal limits. Limited food variety, especially reduced intake of vegetables, fish, and organ meats, may have contributed to these deficiencies (55, 56).

Notably, vitamins K and D remained below recommended levels in both groups post-intervention, with potential implications for bone health, injury risk, and muscle function (57). Vitamin K is found mainly in vegetables and green leafy vegetables (Arugula, Kale, Spinach). Football players did not eat enough from the vegetable group (55). Insufficient consumption of vitamin K may be associated with a raised fracture risk (25). Meanwhile, vitamin D remained low, likely due to limited fish availability. Deficiency is linked to reduced muscle strength and endurance and a higher risk of injuries such as stress fractures (57). These findings align with prior studies reporting consistently low intakes of calcium, vitamin D, and other essential micronutrients among football players (26, 49, 58–61). Therefore, adequate micronutrient intake may warrant attention to ensure players consume the recommended intake of key vitamins and minerals to optimize health and performance.

## 5 Strength and limitations

This study has several advantages that enhance its significance. It provides detailed dietary assessments, offering a multi-faceted perspective and contributing a deeper understanding of the subject. Additionally, the study focuses on elite athletes from the Jordan national football team. This unique aspect enhances the study's relevance by providing insights applicable to high-performance athletes. We have included these advantages in the manuscript to clarify the strengths of our study further. However, the limitations are apparent, too. First, the sample size was relatively small, which may limit the generalizability of the findings to a broader population of football players. Because the study period included both pre- and in-season phases, seasonal differences in energy expenditure and appetite could have influenced dietary intake independently of the intervention. Furthermore, the use of the Harris-Benedict equation to estimate EER. This equation is generally less accurate for athletic populations with high lean body mass. Given that body composition was measured, future research in this population should employ more appropriate prediction equations that incorporate lean mass (e.g., Cunningham equation) for more precise EER determination. Additionally, dietary intake data were self-reported, which may introduce recall significant recall bias and inaccuracies in reported food consumption. When discussing the observed low reported energy intake, it is critical to acknowledge the methodological limitations. The fact that the athletes’ body weight and lean body mass remained consistent throughout the intervention period suggest that they were likely meeting their actual energy needs for weight maintenance, implying that the calculated deficit may be result of measurement errors inherent in self-reported methods or the estimation of energy requirements (Burke, 2018). Performance metrics and perceived exertion (RPE) were not assessed as well. Future research should incorporate physiological and perceptual outcomes to link nutritional intake to functional performance. A final limitation is the lack of a formal assessment of fluid intake or hydration status. Given the intensity of training and the nature of the time-restricted feeding protocol, hydration is a critical factor that could impact thermoregulation overall athletic performance and recovery and should be assessed in future studies using objective markers.

## 6 Conclusions

In conclusion, the 8-week time-restricted feeding (TRF) protocol did not significantly alter energy or macronutrient intake compared to the standard diet (CON). The study highlights a critical, persistent energy and micronutrient deficit in this elite athletic population, emphasizing the need for targeted, mandatory sports nutrition education to mitigate the widespread risk of Relative Energy Deficiency in Sport (RED-S) es.

## Data Availability

The raw data supporting the conclusions of this article will be made available by the authors, without undue reservation.

## Appendix A

(ISSN, 2018).

Adult weight 70 kg, highly active (2.1).

Carbohydrate intake is 7 g/kg equal to 490 g.

Protein intake is 2 g/kg equal to 140 g.

3,500 kcal MEAL PLAN.

**Table:**

	Item	Kcal	Protein (g)	Carbohydrate (g)	Fat (g)
Breakfast	6 tbsp oats	160	6	30	2
	500 mL low-fat milk	240	16	24	10
	100 g strawberries	60	0	15	0
	2 slices whole grain toast	160	6	30	2
	15 g peanut butter	90	0	0	10
	30 g honey	120	0	30	0
Mid morning	2 tbsp raisins	60	0	15	0
	12 almonds	90	0	0	10
	Large banana	120	0	30	0
lunch	200 g sweet potato	160	0	30	2
	10 green olives	45	0	0	5
	5 g olive oil spread	45	0	5	2
	300 g sweet corn	160	6	30	2
	60 g tuna in brine	110	14	0	6
	2 cups green leafy veg	50	4	10	0
Mid afternoon	1 medium apple	60	0	15	0
Post work out	500 mL low-fat milk	240	16	24	10
	1 large banana	120	0	30	0
	30 g honey	120	0	30	0
Dinner	90 g grilled chicken breast	110	14	0	6
	300 g pasta	480	18	90	6
	5 g olive oil	45	0	0	5
	1 cup cooked zucchini	50	4	10	0
	30 g pasta sauce	25	2	5	0
Evening	2 slices whole grain toast	160	6	30	2
	5 g olive oil	45	0	0	5
	1 cup yoghurt	150	8	12	8
	1 egg	75	7	5	5
	30 g avocado	90	0	0	10
Total		3,490	140	490	113
%			16%	56%	28%

## References

[1] Correia JM Santos I Pezarat-Correia P Minderico C Mendonca GV. Effects of intermittent fasting on specific exercise performance outcomes: a systematic review including meta-analysis. Nutrients. (2020) 12(5):1390. 10.3390/nu12051390 32408718 PMC7284994

[2] Nowosad K Sujka M. Effect of various types of intermittent fasting (IF) on weight loss and improvement of diabetic parameters in human. Curr Nutr Rep. (2021) 10:146–54. 10.1007/s13668-021-00353-5 33826120 PMC8102292

[3] Modena A Casiraghi MC Erba D. Dietary intake and adherence to the Mediterranean diet in semi-professional female soccer players: a cross-sectional study. Front Nutr. (2024) 11:1378365. 10.3389/fnut.2024.1378365 38706566 PMC11066240

[4] Langdon S Goedhart E Oosterlaan J Königs M. Heading exposure in elite football (soccer): a study in adolescent, young adult, and adult male and female players. Med Sci Sports Exercise. (2022) 54(9):1459–65. 10.1249/MSS.0000000000002945 PMC9390232 35482757

[5] Aguinaga-Ontoso I Guillen-Aguinaga S Guillen-Aguinaga L Alas-Brun R Guillen-Grima F. Effects of nutrition interventions on athletic performance in soccer players: a systematic review. Life. (2023) 13(6):1271. 10.3390/life13061271 37374054 PMC10301089

[6] Oliveira CC Ferreira D Caetano C Granja D Pinto R Mendes B Nutrition and supplementation in soccer. Sports. (2017) 5(2):28. 10.3390/sports5020028 29910389 PMC5968974

[7] Nédélec M McCall A Carling C Legall F Berthoin S Dupont G. Recovery in soccer: part II—recovery strategies. Sports Med. (2013) 43:9–22. 10.1007/s40279-012-0002-0 23315753

[8] Thomas DT Erdman KA Burke LM. Nutrition and athletic performance. Med Sci Sports Exerc. (2016) 48(3):543–68. 10.1249/MSS.0000000000000852 26891166

[9] Rollo I Williams C. Carbohydrate nutrition and skill performance in soccer. Sports Med. (2023) 53(Suppl 1):7–14. 10.1007/s40279-023-01876-3 37421586 PMC10721660

[10] Collins J Maughan RJ Gleeson M Bilsborough J Jeukendrup A Morton JP UEFA expert group statement on nutrition in elite football. Current evidence to inform practical recommendations and guide future research. Br J Sports Med. (2021) 55(8):416. 10.1136/bjsports-2019-101961 33097528

[11] Kerksick CM Wilborn CD Roberts MD Smith-Ryan A Kleiner SM Jäger R ISSN exercise & sports nutrition review update: research & recommendations. J Int Soc Sports Nutr. (2018) 15(1):38. 10.1186/s12970-018-0242-y 30068354 PMC6090881

[12] Burke LM Loucks AB Broad N. Energy and carbohydrate for training and recovery. J Sports Sci. (2006) 24(7):675–85. 10.1080/02640410500482602 16766497

[13] Jäger R Kerksick CM Campbell BI Cribb PJ Wells SD Skwiat TM International society of sports nutrition position stand: protein and exercise. J Int Soc Sports Nutr. (2017) 14(1):20. 10.1186/s12970-017-0177-8 28642676 PMC5477153

[14] Tsameret S Chapnik N Froy O. Effect of early vs. late time-restricted high-fat feeding on circadian metabolism and weight loss in obese mice. Cell Mol Life Sci. (2023) 80(7):180. 10.1007/s00018-023-04834-4 37329359 PMC11072437

[15] Brady AJ Langton HM Mulligan M Egan B. Effects of 8 wk of 16: 8 time-restricted eating in male middle-and long-distance runners. Med Sci Sports Exercise. (2021) 53(3):633–42. 10.1249/MSS.0000000000002488 32796255

[16] Burke LM Hawley JA Wong SHS Jeukendrup AE. Carbohydrates for training and competition. J Sports Sci. (2011) 29(sup1):S17–27. 10.1080/02640414.2011.585473 21660838

[17] Kreider RB Wilborn CD Taylor L Campbell B Almada AL Collins R ISSN Exercise & Sport Nutrition Review: Research & Recommendations. New York, NY: Springer (2010). p. 7.

[18] Montero-López E Santos-Ruiz A García-Ríos MC Rodríguez-Blázquez M Rogers HL Peralta-Ramírez MI. The relationship between the menstrual cycle and cortisol secretion: daily and stress-invoked cortisol patterns. Int J Psychophysiol. (2018) 131:67–72. 10.1016/j.ijpsycho.2018.03.021 29605399

[19] Cook CJ Fourie P Crewther BT. Menstrual variation in the acute testosterone and cortisol response to laboratory stressors correlate with baseline testosterone fluctuations at a within-and between-person level. Stress. (2021) 24(4):458–67. 10.1080/10253890.2020.1860937 33287617

[20] von Hurst PR Walsh DCI Conlon CA Ingram M Kruger R Stonehouse W. Validity and reliability of bioelectrical impedance analysis to estimate body fat percentage against air displacement plethysmography and dual-energy X-ray absorptiometry. Nutr Diet. (2016) 73(2):197–204. 10.1111/1747-0080.12172

[21] Fornetti WC Pivarnik JM Foley JM Fiechtner JJ. Reliability and validity of body composition measures in female athletes. J Appl Physiol. (1999) 87(3):1114–22. 10.1152/jappl.1999.87.3.1114 10484585

[22] Spehnjak M Gušić M Molnar S Baić M Andrašić S Selimi M Body composition in elite soccer players from youth to senior squad. Int J Environ Res Public Health. (2021) 18(9):4982. 10.3390/ijerph18094982 34067121 PMC8125322

[23] Potgieter S. Sport nutrition: a review of the latest guidelines for exercise and sport nutrition from the American college of sport nutrition, the international Olympic committee and the international society for sports nutrition. S Afr J Clin Nutr. (2013) 26(1):6–16. 10.1080/16070658.2013.11734434

[24] Sebastiá-Rico J Soriano JM Sanchis-Chordà J Alonso-Calvar M López-Mateu P Romero-García D Dietary habits of elite soccer players: variations according to competitive level, playing position and sex. Nutrients. (2023) 15(20):1–14. 10.3390/nu15204323 PMC10609682 37892399

[25] Hrubša M Siatka T Nejmanová I Vopršalová M Kujovská Krčmová L Matoušová K Biological properties of vitamins of the B-complex, part 1: vitamins B1, B2, B3, and B5. Nutrients. (2022) 14(3):484. 10.3390/nu14030484 35276844 PMC8839250

[26] Książek A Zagrodna A Słowińska-Lisowska M. Assessment of the dietary intake of high-rank professional male football players during a preseason training week. Int J Environ Res Public Health. (2020) 17(22):8567. 10.3390/ijerph17228567 33218191 PMC7699180

[27] Bettonviel AEO Brinkmans NYJ Russcher K Wardenaar FC Witard OC. Nutritional status and daytime pattern of protein intake on match, post-match, rest and training days in senior professional and youth elite soccer players. Int J Sport Nutr Exerc Metab. (2016) 26(3):285–93. 10.1123/ijsnem.2015-0218 26630203

[28] Raizel R Da Mata Godois A Coqueiro AY Voltarelli FA Fett CA Tirapegui J Pre-season dietary intake of professional soccer players. Nutr Health. (2017) 23(4):215–22. 10.1177/0260106017737014 29037118

[29] Brinkmans NYJ Iedema N Plasqui G Wouters L Saris WHM van Loon LJC Energy expenditure and dietary intake in professional football players in the Dutch premier league: implications for nutritional counselling. J Sports Sci. (2019) 37(24):2759–67. 10.1080/02640414.2019.1576256 30773995

[30] Iglesias-Gutiérrez E García Á García-Zapico P Pérez-Landaluce J Patterson ÁM García-Rovés PM. Is there a relationship between the playing position of soccer players and their food and macronutrient intake? Appl Physiol Nutr Metab. (2012) 37(2):225–32. 10.1139/h11-152 22380725

[31] Rico-Sanz J Frontera WR Molé PA Rivera MA Rivera-Brown A Meredith CN. Dietary and performance assessment of elite soccer players during a period of intense training. Int J Sport Nutr Exerc Metab. (1998) 8(3):230–40. 10.1123/ijsn.8.3.230 9738133

[32] Giada F Zuliani G Baldo-Enzi G Palmieri E Volpato S Vitale E Lipoprotein profile, diet and body composition in athletes practicing mixed an anaerobic activities. J Sports Med Phys Fitness. (1996) 36(3):211–6. 8979651

[33] Jacobs I Westlin N Karlsson J Rasmusson M Houghton B. Muscle glycogen and diet in elite soccer players. Eur J Appl Physiol Occup Physiol. (1982) 48:297–302. 10.1007/BF00430219 7200872

[34] Bangsbo J Nørregaard L Thorsøe F. The effect of carbohydrate diet on intermittent exercise performance. Int J Sports Med. (1992) 13(2):152–7. 10.1055/s-2007-1021247 1555905

[35] Hassapidou MN Grammatikopoulou MG Liarigovinos T. Dietary intakes of Greek professional football players. Nutr Food Sci. (2000) 30(4):191–4. 10.1108/00346650010330234

[36] Schubert MM Sabapathy S Leveritt M Desbrow B. Acute exercise and hormones related to appetite regulation: a meta-analysis. Sports Med. (2014) 44:387–403. 10.1007/s40279-013-0120-3 24174308

[37] Caruana Bonnici D Akubat I Greig M Sparks A Mc Naughton LR. Dietary habits and energy balance in an under 21 male international soccer team. Res Sports Med. (2018) 26(2):168–77. 10.1080/15438627.2018.1431537 29366354

[38] Mountjoy M Sundgot-Borgen J Burke L Ackerman KE Blauwet C Constantini N International Olympic committee (IOC) consensus statement on relative energy deficiency in sport (RED-S): 2018 update. Int J Sport Nutr Exerc Metab. (2018) 28(4):316–31. 10.1123/ijsnem.2018-0136 29771168

[39] Pensgaard AM Sundgot-Borgen J Edwards C Jacobsen AU Mountjoy M. Intersection of mental health issues and relative energy deficiency in sport (REDs): a narrative review by a subgroup of the IOC consensus on REDs. Br J Sports Med. (2023) 57(17):1127–35. 10.1136/bjsports-2023-106867 37752005

[40] Staśkiewicz W Grochowska-Niedworok E Zydek G Białek-Dratwa A Grajek M Jaruga-Sȩkowska S Changes in body composition during the macrocycle of professional football players in relation to sports nutrition knowledge. Front Nutr. (2022) 9:981894. 10.3389/fnut.2022.981894 36523334 PMC9745111

[41] Martín-Rodríguez A Belinchón-deMiguel P Rubio-Zarapuz A Tornero-Aguilera JF Martínez-Guardado I Villanueva-Tobaldo CV Advances in understanding the interplay between dietary practices, body composition, and sports performance in athletes. Nutrients. (2024) 16(4):1–32. 10.3390/nu16040571 PMC10892519 38398895

[42] Heaney S O’Connor H Naughton G Gifford J. Towards an understanding of the barriers to good nutrition for elite athletes. Int J Sports Sci Coach. (2008) 3(3):391–401. 10.1260/174795408786238542

[43] Rodriguez NR Di Marco NM Langley S. American college of sports medicine position stand. Nutrition and athletic performance. Med Sci Sports Exerc. (2009) 41(3):709–31. 10.1249/MSS.0b013e31890eb86 19225360

[44] Devlin BL Leveritt MD Kingsley M Belski R. Dietary intake, body composition, and nutrition knowledge of Australian football and soccer players: implications for sports nutrition professionals in practice. Int J Sport Nutr Exerc Metab. (2017) 27(2):130–8. 10.1123/ijsnem.2016-0191 27710165

[45] Anderson L Orme P Naughton RJ Close GL Milsom J Rydings D Energy intake and expenditure of professional soccer players of the English premier league: evidence of carbohydrate periodization. Int J Sport Nutr Exerc Metab. (2017) 27(3):228–38. 10.1123/ijsnem.2016-0259 28050927

[46] Andrews MC Itsiopoulos C. Room for improvement in nutrition knowledge and dietary intake of male football (soccer) players in Australia. Int J Sport Nutr Exerc Metab. (2016) 26(1):55–64. 10.1123/ijsnem.2015-0064 26251549

[47] Mata F Valenzuela PL Gimenez J Tur C Ferreria D Domínguez R Carbohydrate availability and physical performance: physiological overview and practical recommendations. Nutrients. (2019) 11(5):1084. 10.3390/nu11051084 31100798 PMC6566225

[48] Urhan M Yıldız H. Assessment of diet quality and nutrition status of Turkish elite adolescent male soccer players. Spor Bilim Derg. (2022) 33(1):19–31. 10.17644/sbd.954537

[49] Jenner SL Buckley GL Belski R Devlin BL Forsyth AK. Dietary intakes of professional and semi-professional team sport athletes do not meet sport nutrition recommendations—a systematic literature review. Nutrients. (2019) 11(5):1160. 10.3390/nu11051160 31126159 PMC6567121

[50] Kirwan RD Kordick LK McFarland S Lancaster D Clark K Miles MP. Dietary, anthropometric, blood-lipid, and performance patterns of American college football players during 8 weeks of training. Int J Sport Nutr Exerc Metab. (2012) 22(6):444–51. 10.1123/ijsnem.22.6.444 22805315

[51] Abbey EL Wright CJ Kirkpatrick CM. Nutrition practices and knowledge among NCAA division III football players. J Int Soc Sports Nutr. (2017) 14(1):13. 10.1186/s12970-017-0170-2 28529463 PMC5437483

[52] Thomas DT Erdman KA Burke LM. Position of the academy of nutrition and dietetics, dietitians of Canada, and the American college of sports medicine: nutrition and athletic performance. J Acad Nutr Diet. (2016) 116(3):501–28. 10.1016/j.jand.2015.12.006 26920240

[53] Williams C Rollo I. Carbohydrate nutrition and team sport performance. Sports Med. (2015) 45:13–22. 10.1007/s40279-015-0399-3 PMC4672015 26553494

[54] Ghazzawi HA Hussain MA Raziq KM Alsendi KK Alaamer RO Jaradat M Exploring the relationship between micronutrients and athletic performance: a comprehensive scientific systematic review of the literature in sports medicine. Sports. (2023) 11(6):109. 10.3390/sports11060109 37368559 PMC10302780

[55] Lukaski HC. Vitamin and mineral status: effects on physical performance. Nutrition. (2004) 20(7–8):632–44. 10.1016/j.nut.2004.04.001 15212745

[56] Rockwell MS Kostelnik SB McMillan RP Lancaster M Larson-Meyer DE Hulver MW. An association between bioavailable 25-hydroxyvitamin D and bone mineral density in a diverse cohort of collegiate athletes. Med Sci Sports Exerc. (2022) 54(3):371–6. 10.1249/MSS.0000000000002807 34652336

[57] Chryssanthopoulos C Souglis A Tsalouhidou S Hulton AT Bogdanis GC Petridou A Dietary intake of soccer players before, during and after an official game: influence of competition level and playing position. Nutrients. (2024) 16(3):337. 10.3390/nu16030337 38337622 PMC10856869

[58] Hidalgo R Elizondo T Bermudo FMM Méndez RP Amorós GB Padilla EL Nutritional intake and nutritional status in elite Mexican teenagers soccer players of different ages. Nutr Hosp. (2015) 32(4):1735–43. 26545544 10.3305/nh.2015.32.4.8788

[59] Abdelhaliem HS El Gezrey HM. Evaluation of nutrition intake of football players. J Med Sci Res. (2018) 1(1):10. 10.4103/JMISR.JMISR_13_18

[60] McCrink CM McSorley EM Grant K McNeilly AM Magee PJ. An investigation of dietary intake, nutrition knowledge and hydration status of Gaelic football players. Eur J Nutr. (2021) 60:1465–73. 10.1007/s00394-020-02341-x 32734346 PMC7987599

[61] Burke LM, Lundy B, Fahrenholtz IL, Melin AK. Pitfalls of conducting and interpreting estimates of energy availability in free-living athletes. Int J Sport Nutr Exerc Metab. 28(4):350–63. 10.1123/ijsnem.2018-0142 30029584

